# Psychosocial Predictors of Metabolic Syndrome among Latino Groups in the Multi-Ethnic Study of Atherosclerosis (MESA)

**DOI:** 10.1371/journal.pone.0124517

**Published:** 2015-04-23

**Authors:** Manuel S. Ortiz, Hector F. Myers, Christine Dunkel Schetter, Carlos J. Rodriguez, Teresa E. Seeman

**Affiliations:** 1 Department of Psychology, Universidad de La Frontera, Temuco, Chile; 2 Center for Medicine, Health & Society and Psychology, Vanderbilt University, Nashville, Tennessee, United States of America; 3 Department of Psychology, University of California Los Angeles, Los Angeles, California, United States of America; 4 Division of Public Health Sciences, Department of Medicine, Wake Forest University School of Medicine, Winston-Salem, North Carolina, United States of America; 5 David Geffen School of Medicine, University of California Los Angeles, Los Angeles, California, United States of America; Steno Diabetes Center, DENMARK

## Abstract

**Objective:**

We sought to determine the contribution of psychological variables to risk for metabolic syndrome (MetS) among Latinos enrolled in the Multi-Ethnic Study of Atherosclerosis (MESA), and to investigate whether social support moderates these associations, and whether inflammatory markers mediate the association between psychological variables and MetS.

**Research design and methods:**

Cross-sectional analyses at study baseline were conducted with a national Latino cohort (n = 1,388) that included Mexican Americans, Dominican Americans, Puerto Rican Americans and Central/South Americans. Hierarchical logistic regression analyses were conducted to test the effects of psychosocial variables (chronic stress, depressive symptoms, and social support) on MetS. In addition, separate subgroup-specific models, controlling for nationality, age, gender, socioeconomic position, language spoken at home, exercise, smoking and drinking status, and testing for the effects of chronic stress, depressive symptoms and inflammation (IL-6, CRP, fibrinogen) in predicting risk for MetS were conducted.

**Results:**

In the overall sample, high chronic stress independently predicted risk for MetS, however this association was found to be significant only in Mexican Americans and Puerto Rican Americans. Social support did not moderate the associations between chronic stress and MetS for any group. Chronic stress was not associated with inflammatory markers in either the overall sample or in each group.

**Conclusions:**

Our results suggest a differential contribution of chronic stress to the prevalence of MetS by national groups.

## Introduction

Metabolic syndrome (MetS) represents the cluster of metabolic abnormalities associated with an increased risk of diabetes mellitus and cardiovascular disease. The Hispanic/Latino population living in the United States has the highest prevalence rate of MetS compared to other ethnics groups [1]. They also carry a disproportionate burden of related risk factors (e.g. overweight & obesity) and disease sequelae (i.e. diabetes & hypertension) [2,3].

The U.S. Latino population includes diverse groups, such as Dominican Americans, Puerto Rican Americans, Mexican Americans, Cuban Americans, as well as Central and South Americans. Although they are commonly thought of as a single monolithic ethnic group, there are substantial sociocultural differences between the groups, including differences in health risks that are likely the result of the known cultural, genetic and socioeconomic heterogeneity of this population [4]. Furthermore, genetic admixture studies have established that Mexican-origin Hispanics are predominantly of European and Native American ancestry, that Caribbean-Hispanics (Dominicans, Cubans and Puerto Ricans) are mainly of European and African ancestry, and that most of the South and Central American-Hispanics share European, Native American and African ancestry, but in different proportions [5,6]. Therefore, studies that include Latino groups should not only compare them with other ethnic groups, but also identify within-group differences in health-relevant variables.

A previous study by Allison et al. [4] with the Latino sample enrolled in the Multi-Ethnic Study of Atherosclerosis (MESA) identified differences in cardiovascular disease risks, and different mean values on several indicators of subclinical cardiovascular disease in the four U.S. Hispanic subgroups. Specifically, Mexican Americans had the highest prevalence of MetS and triglycerides concentrations compared to Dominican Americans, Puerto-Rican Americans and other Central/South Americans. However, no differences in fasting glucose, systolic blood pressure, low LDL cholesterol, and inflammatory markers such as C-reactive protein and fibrinogen were noted.

Our study expands on the Allison et al. study [4] by testing the contribution of psychological factors on risk for MetS in these four groups. To our knowledge, no population-based study has examined the association between the burden of stress, depressive symptoms, and psychosocial resources in predicting the onset of metabolic syndrome among the U.S. Latino subgroups, nor have they investigated any between-group differences in the pattern of relationships among these variables. Previous studies have identified the links between psychosocial variables and MetS but mainly in Caucasian populations [7–11]. According to the extant literature, the odds of developing MetS increases 13% for those who experience negative life events [12], and employees with chronic stress at work are twice as likely to develop MetS than those without chronic work stress [13]. Similarly, many studies have found that depressive disorder is related to incident and/or recurrent cardiovascular disease in both patients with cardiovascular disease and in the general population [14]. In addition, both cross-sectional [15] and longitudinal associations [16] between depressive symptoms and MetS have been reported. On the other hand, psychosocial resources, such as the availability of family social support, have been linked to more positive mental and physical health [17,18]. Loneliness (as an indirect measure of lack of social support) was associated with an increased likelihood of meeting MetS criteria [19], and MetS and its components were more prevalent in those with low social support, as well as in men and women with high rates of dyslipidemia [20].

Systemic inflammation has emerged as an important cardiovascular risk factor, and may be an underlying mechanism linking psychosocial factors to cardiovascular disease [21]. Inflammatory processes have been linked to the onset and development of atherosclerosis, and with the precipitation of cardiovascular disease [22]. Evidence obtained from the MESA study has suggested that psychosocial factors are related to higher levels of inflammatory markers. For example, higher levels of chronic stress were associated with higher concentrations of IL-6 and CRP, and depressive symptoms were also positively associated with levels of IL-6. However, these effects were reduced to non-significance after controlling for socioeconomic position (SEP), BMI, health risk behaviors, and diabetes [21].

To date, most studies supporting the link between psychological factors and MetS have been conducted with Caucasians or by treating race/ethnicity as a covariate. This limits the generalizability of the findings, and fails to consider any between and within-group differences in the pattern of associations between psychological factors and MetS. Our study addresses these limitations by testing the association of psychological variables of stress and depressive symptoms with risk for MetS in a multi-national sample of Latinos living in the US. Thus, the purpose of this study was to test the contribution of psychological variables to risk for MetS among Latinos enrolled in the Multi-Ethnic Study of Atherosclerosis, testing associations in both the overall sample of Latinos and in each Latino group. We also investigate whether social support moderates, and whether inflammation mediated the association between psychological variables and MetS.

## Materials and Methods

The Multi-Ethnic Study of Atherosclerosis (MESA) is a population-based prospective cohort study of 6,814 US adults from different racial/ethnic groups. Data were collected from several university study centers, including: Wake Forest, Columbia, John Hopkins (city and county tracts), Minnesota, Northwestern, and UCLA. The current, cross-sectional analyses focus only on data on the Latino subsample from the baseline MESA exam (July 2000—July 2002).

### Ethical Statement

The institutional review boards at all participating study centers approved the protocol, and all participants gave informed written consent. Patient records/information was de-identified prior to analysis. MESA was reviewed by Institutional Review Boards at all six field centers and the Coordinating Center: Columbia University (Field Center); Johns Hopkins University (Field Center); Northwestern University (Field Center); UCLA (Field Center); University of Minnesota (Field Center); Wake Forest University (Field Center); University of Washington (Coordinating Center). Details about the study have been previously published [23].

### Participants

Analyses were conducted only on the MESA Latino sample (n = 1388), which includes: Mexican Americans (n = 799), Dominican Americans (n = 175), Puerto Rican Americans (n = 201), and other Central/South Americans (n = 213). Participants with known cardiovascular disease were excluded at baseline.

### Measures

#### Explanatory variables

Chronic stress was assessed with the Chronic Stress Burden Scale that was developed for the Healthy Women’s Study [24]. Participants were asked to identify 5 ongoing difficulties, and report if this has been a problem for six months or more (“*health problem*”, “*health problem in someone close to them*”, “*job difficulties*”, “*financial strains*”, and “*difficulties in a relationship with someone close*”). Items were dichotomous (0 = “no”, 1 = “yes”) and a total score was calculated by summing the total number of items to which a “yes” response was given (range 0 to 5).

Depressive symptoms were measured with the Center for Epidemiologic Studies Depression scale (CES-D) [25], which is a 20-item self-report depression symptoms measure that was rated on a 0 to 3 point scale (0 = “rarely”; 3 = “most”). The CES-D assesses depressed mood (“*past week*, *I felt sad*”), feelings of worthlessness (“*past week*, *I felt I was not as good as other people*”), feelings of hopelessness (“*past week I felt hopeful about future*”), poor concentration (“*past week*, *I had trouble keeping my mind on what I am doing*”), loss of appetite (“*past week*, *I had poor appetite*”), and sleep disturbance (“*past week*, *sleep was restless*”). High scores are indicative of greater depression. In this study, we used the scores as a continuous variable (Cronbach α = .88).

Social support was assessed with the ENRICHD Social Support Inventory (ESSI) [26]. The original scale is a 7-item self-report measure that assesses structural, instrumental, and emotional support. Samples items are “*someone is available to help with daily chores*”, or “*someone is available to provide emotional support*”. Responses were rated on a five-point scale (1 = “none of the time”; 5 = “all of the time”), except for the seventh item, which has a yes/no response. In MESA, participants responded only to 6 items and the dichotomous item was excluded. The Cronbach Alpha for the scale was. 88.

#### Outcomes

The primary study outcome was Metabolic Syndrome as defined by the criteria proposed by the National Cholesterol Education Program’s Adult Treatment Panel III IDF in 2004 [27]. In this study MetS was treated as a dichotomous variable (MetS or not MetS). According to the definition, in order to be diagnosed with MetS, participants must meet 3 or more of the following criteria:

1. Increased waist size: > 102 cm for males; > 88 cm for females
2. Elevated triglycerides: ≥ 150 mg/dl
3. Low HDL cholesterol: < 40 mg/dl for males; < 50 mg/dl for females
4. Elevated fasting glucose level (IFG): ≥ 110 mg/dl, or being treated for diabetes.
5. Elevated blood pressure: Systolic blood pressure ≥ 130; Diastolic blood pressure ≥ 85; or being treated for hypertension.

Triglycerides and HDL cholesterol were measured from blood samples obtained after 12 hour fasting. Serum glucose was measured by the glucose oxidase method on the Vitros analyzer (Johnson & Johnson Clinical Diagnostics). Resting blood pressure was measured three times in the seated position using a Dinamap model Pro 100 automated oscillometric sphygmomanometer (Critikon, Tampa, Florida). The average of the last two measurements was used in the analyses [28].

#### Covariates

Demographic characteristics: Several demographic characteristics were included in the analyses: Latino sub-group containing four categories (Mexican American, Dominican American, Puerto Rican American, and Central/South American), educational attainment, income, language spoken at home, years living in US, age and sex. Education and income scores were standardized and summed to create a socioeconomic position (SEP) index [29].

Health behaviors: Standard questionnaires were administered to measure several health behaviors. *Cigarette smoking* categories were never (0), former (1), and current (2). Never smoking was defined as lifetime consumption of less than 100 cigarettes; and former smoking as quit smoking ≥ 1 year earlier. Alcohol use was screened with the questions *“have you ever consumed alcoholic beverages?”* and *“do you presently drink alcoholic beverages?”* Participants were classified as never (0), former (1), and current (2) users [30].

Physical activity was measured with the intentional exercise variable from the Typical Week Physical Activity Survey, defined as the sum of walking for exercise, sports/dancing, and conditioning metabolic equivalent (MET) hours per week [23].

For analyses purposes, U.S. Latino group, gender, language spoken at home, cigarette smoking and alcohol use were dummy coded, with Mexican American, female, Spanish spoken at home, non-smoker status, and current drinker selected as the reference for each variable. These variables were included in our analyses as covariates.

Inflammatory markers: Interleukin 6 (IL-6), CRP (C reactive protein) and fibrinogen, were collected using standard procedures [21]. None of the inflammatory variables distributed normally so they were log transformed. IL-6 level was measured with an ultrasensitive enzyme-linked immunoabsorbent assay (R&D Systems Minneapolis, Minnesota). The laboratory coefficient of variation for the IL-6 assay was 6.3%; CRP and fibrinogen antigen levels were measured using the BNII nephelometer (N high sensitivity to CRP and N antiserum to human fibrinogen; Dade-Behring, San Mateo, CA) [21]. The intra-assay coefficient of variation for CRP ranged from 2.3% to 4.4%, and inter-assay coefficients of variation ranged from 2.1 to 5.7%. The intra-assay and inter-assay coefficients of variation for fibrinogen were 2.7 and 26%, respectively [30].

### Data analysis

Data were analyzed with SPSS version 21. All the analyses are cross-sectional, focusing on data from exam 1. Hierarchical logistic regression was conducted to estimate the contribution of variables of interest in predicting risk for MetS in the overall Latino sample. These models included variables entered in invariant order as follows: *Step 1* included demographic characteristics (age, socioeconomic position, language spoken at home, years living in US, Latino sub-group, and gender), and lifestyle behaviors (smoking history, alcohol consumption, and physical activity level); *Step 2* added psychosocial variables: chronic stress, depressive symptoms, and social support; *Step 3* added the interaction terms between each psychosocial variable and national groups (chronic stress X national group, depressive symptoms X national group); and *Step 4* added the inflammatory markers (IL-6, CRP, and fibrinogen levels).

If the interaction terms between psychosocial variables and national groups are not statistically significant were dropped from the analyses. Thus, additional subgroup-specific hierarchical logistic regressions were conducted to determine the association between psychological factors and risk for MetS in each Latino group sampled. These models included age, socioeconomic position, language spoken at home, gender, smoking history, alcohol consumption, and physical activity level, and inflammatory markers.

For all the analyses a p-value less than 0.05 was considered statistically significant.

## Results

Table 1 shows the sociodemographic characteristics at baseline for each of the four U.S. Hispanic groups, and for the total sample (n = 1388). The mean age was 60.85 years (SD = 10.2) and 52% were female. Fifty-eight percent were Mexican American, 12.6% were Dominican American, 14.5% were Puerto Rican American, and 15.3% were Central/South American. Mexican Americans were the oldest group (M = 61.47; SD = 10.3), and Dominican Americans had lived the longest in the United States (M = 41.29; SD = 13.1). Sixty-eight percent of the sample was born outside of the United States. Ninety-five percent of the Central/South Americans were born outside the Unites States (16.9% from El Salvador, 14.1% from Ecuador, 11.3% from Colombia, 10.8% from Guatemala, and 7.5% from Peru). Sixty-percent of the sample was married, and 67.2% spoke Spanish at home. Thirty four percent completed grade 8 or less of education, and 80.1% had an annual income equal to or less than $50,000.

**Table 1:** Demographics characteristics.

	Mexican American	Dominican American	Puerto Rican American	Central/South American	Total	p
	(n = 799)	(n = 175)	(n = 201)	(n = 213)	(n = 1388)	
Age (mean, SD)	61.47(10.3)	59.05(10.2)	59.65(10.1)	61.09(10.2)	60.85(10.2)	.011
Years lived in USA (mean, SD)	28.02(16.9)	25.83(10.7)	41.29(13.1)	25.42(12.6)	29.43(15.4)	.001
MetS women%	57	44.3	42.2	44.3	50.9	.001
MetS men%	43.4	23.1	34.8	38.5	39.2	.001
Male %	50.4	44.6	45.8	42.7	47.8	.099
US born%	50.4	0	16.9	5.2	32.2	.001
Married%	60.6	61.7	55.7	61	60.1	.95
Education %						
* Grade 8 or less*	37.5	40	17.9	31	34	.01
* Bachelor*	4.4	4.6	8	7.5	5.4	.46
* Graduate or professional*	2.9	6.3	3	7.5	4	.18
Income %						
< $50000	79.2	84	78.6	81.7	80.1	.45

Hierarchical logistic regressions were conducted to estimate the relative contributions of the variables of interest in predicting risk for MetS, and considered Mexican American nationality, female gender, Spanish spoken at home, non-smoker status, and current drinker as the reference group for each of these categorical variables.

Table 2 shows the odds ratio and confidence intervals for each predictor. Step 1 included sociodemographic variables, and lifestyle behaviors. Mexican Americans had the highest prevalence of MetS. The odds ratio for MetS was 0.53 (95% CI = 0.37, 0.77) for Dominican Americans compared to the Mexican Americans, 0.64 (95% CI = 0.46, 0.89) for Puerto Rican Americans, and 0.67 (95% CI = 0.48, 0.93) for the Central/South-Americans. Males had lower risk of having MetS than females (OR = 0.62; 95% CI = 0.49, 0.80). As expected, older age was associated with greater risk of having MetS (OR = 1.03, 95% CI = 1.01, 1.04), and higher socioeconomic position was a protective factor (OR = 0.93; 95% CI = 0.86, 0.96). Language spoken at home, exercise and smoking history were not significantly related to risk of MetS, but current drinkers (reference group) were 47% more likely to have MetS compared to former drinkers (OR = 1.47; 95% CI = 1.11, 1.95). Further, adding the psychosocial factors to the model (chronic stress, depressive symptoms, and social support) did not alter these findings (see Step 2, Table 2). The odds ratio for chronic stress was 1.12 (95%CI = 1.01, 1.23), indicating that the risk of being diagnosed with MetS increases by 12% for each unit increase in chronic stress. Neither depressive symptoms nor social support were related to prevalence of MetS. The interaction terms included in step 3 (chronic stress X national group, depressive symptoms X national group) were dropped since none were statistically significant. Adding the inflammatory markers did not change these findings.

**Table 2:** Predictors for MetS risk.

	Step 1		Step 2a		Step 3	
	OR	CI 95%	OR	CI 95%	OR	CI 95%
Dominican American	0.53	0.37, 0.77	0.53	0.37, 0.76	0.64	0.44, 0.94
Puerto Rican American	0.64	0.46, 0.89	0.64	0.46, 0.90	0.64	0.45, 0.90
Central/South American	0.67	0.48, 0.93	0.66	0.47, 0.91	0.70	0.50, 0.98
Male	0.62	0.49, 0.80	0.61	0.47, 0.80	0.73	0.55, 0.96
English spoken at home	0.93	0.69, 1.25	0.98	0.73, 1.33	1.07	0.785, 1.45
Age	1.03	1.01, 1.04	1.03	1.01, 1.04	1.02	1.01, 1.04
SEP	0.93	0.86, 0.96	0.91	0.85, 0.98	0.91	0.85, 0.99
Physical activity	1.00	1.00, 1.00	1.00	1.00, 1.00	1.00	1.00, 1.00
Former smoker *	0.83	0.58, 1.20	0.83	0.57, 1.20	0.91	0.62, 1.33
Current smoker *	0.94	0.64, 1.38	0.92	0.63,1.40	0.95	0.64,1.41
Never drinker †	1.30	0.95, 1.77	1.30	0.96, 1.80	1.31	0.95, 1.81
Former drinker †	1.47	1.11, 1.95	1.49	1.22, 1.97	1.44	1.08, 1.93
Chronic stress			1.12	1.01, 1.23	1.11	1.01, 1.24
Depressive symptoms			0.98	0.97, 0.99	0.98	0.97, 1.00
Emotional support			0.99	0.97, 1.01	0.99	0.96, 1.01
IL-6					3.02	1.80, 5.08
CRP					2.11	1.51, 2.93
Fibrinogen					0.69	0.13, 3.57

* Non-smoker status was used as reference group.

† Current drinker was used as reference group.

Table 3 depicts the association between psychological factors and MetS by Latino subgroup, over and above demographic variables and inflammatory markers. The odds ratio for chronic stress predicting MetS was statistically significant for Mexican—Americans, and Puerto Ricans. Although the OR for stress predicting MetS was greater for Puerto Ricans 1.33 (CI95% = 1.00, 1.75) than for Mexican Americans 1.21 (CI95% 1.04, 1.42), these OR were not statistically significantly different, likely as a function of the significantly smaller sample size for the Puerto Ricans vs. the Mexican-Americans.

**Table 3:** Fully adjusted model for each national group.

	Mexican-American (n = 799)		Dominican-American (n = 175)		Puerto Rican-American (n = 201)		Central/South American (n = 213)	
Predictors	OR	CI95%	OR	CI95%	OR	CI95%	OR	CI95%
Age	1.03	1.01, 1.04	1.02	0.97, 1.08	1.05	1.00, 1.09	1.00	0.97, 1.05
Male	0.73	0.51, 1.06	0.49	0.19, 1.24	1.11	0.51, 2.38	0.98	0.42, 2.30
SEP	0.99	0.88, 1.12	0.78	0.59, 1.06	0.93	0.71, 1.22	0.79	0.61, 1.02
English spoken at home	1.33	0.85, 2.03	0.79	0.18, 3.45	0.61	0.26, 1.43	1.01	0.42, 2.42
Never drinker	1.03	0.65, 1.65	0.93	0.27, 3.17	2.04	0.74, 5.60	0.80	0.30, 2.14
Former drinker	0.73	0.47, 1.12	1.08	0.37, 3.09	1.09	0.44, 2.69	0.38	0.14, 0.99
Former smoker	1.19	0.83, 1.70	1.13	0.39, 3.23	0.49	0.19, 1.21	0.89	0.39, 2.06
Current smoker	1.36	0.82, 2.27	0.36	0.89, 1.42	0.97	0.39, 2.42	0.72	0.22, 2.33
Chronic stress	1.21	1.04, 1.42	0.97	0.64, 1.48	1.33	1.00, 1.75	1.12	0.82, 1.51
Depressive symptoms	0.98	0.98, 1.04	0.96	0.91, 1.01	0.99	0.95, 1.04	0.99	0.95, 1.03
Social Support	1.01	0.98, 1.04	0.98	0.90, 1.06	0.96	0.91, 1.04	1.00	0.94, 1.06
IL-6	1.44	1.07, 1.96	4.60	1.86, 11.4	1.44	0.77, 2.73	1.82	0.89, 3.73
CRP	1.38	1.14, 1.68	1.36	0.89, 2.07	1.60	1.02, 2.50	1.38	0.87, 2.19
Fibrinogen	0.55	0.21, 1.46	0.38	0.03, 4.76	1.66	0.19, 14.9	7.77	0.99, 60.3

Additional analyses were conducted adding an interaction term for chronic stress and social support to test whether social support moderated the relationship between chronic stress and risk for MetS, however it was not statistically significant. Also, chronic stress was not statistically associated with any of the inflammatory markers in either the overall sample or in each group, thus ruling out a role as a potential mediator in this data

## Discussion

The aims of this study were: (1) To test the contribution of psychological variables to risk for MetS in both the overall Latino sample as well as in each Latino subgroup enrolled in MESA; (2) To determine if social support moderates these associations: (3) To assess whether inflammatory markers mediate the associations between psychological variables and MetS. Our findings suggest an increased risk for MetS among participants who reported high levels of chronic stress, independent of sociodemographic characteristics and lifestyle behaviors. Those participants reporting greater chronic stress had significantly higher risk for MetS, and this relationship remained significant after accounting for level of inflammatory markers. This finding is consistent with previous studies that have suggested that exposure to greater chronic stress may increase the odds of developing MetS [13] and adds by demonstrating this association among the U.S. Latinos. Contrary to expectations, depressive symptoms were not related to prevalence of MetS in this sample. Although some studies have reported a positive relationship between depressive symptoms and MetS [16], another study did not find that depressive symptoms at baseline were related with incident hypertension (one criteria for MetS diagnosis) after two years of follow-up [31]. Exploring possible explanations, we found that the distribution for depressive symptoms was negatively skewed, with the median lower than the mean and the mode was zero producing restriction of range. As such, the relationship between depressive symptoms and MetS merits further attention.

Interestingly, when running separate analyses by Latino groups the pattern of association emerges only for the Mexican Americans and Puerto Rican Americans; that is, chronic stress was associated with risk for MetS only in these two groups, suggesting that the role of psychological stress as a risk factor for developing MetS may vary in the different Latino groups. In line with our findings, a study conducted in The Hispanic Community Health Study / Study of Latinos revealed that the prevalence of diabetes was higher for Mexican Americans and Puerto Ricans Americans in comparison with Cubans Americans and South Americans [32]. Furthermore, previous studies testing for within-groups differences in health-relevant variables have found that the prevalence of psychiatric disorders, especially depressive and anxiety disorders, and substance abuse disorders is different between Latino groups [33,34]. Therefore, several explanations for this finding are worth consideration, including that the diet of Mexican Americans deteriorates with acculturation [35]. In particular, studies demonstrated that the more acculturated Mexican Americans replace their traditional healthful dietary practice with more frequent intake of processed food, fast food, more saturated fat, fewer fruits and vegetables, and lower dietary fiber [36–39]. Moreover, Puerto Ricans evidence the highest prevalence of psychiatric disorders, higher levels of unemployment in comparison to others Latinos studied, and may feel more discriminated against than foreign-born Latinos because they do not enjoy the socioeconomic advantages enjoyed by the majority of non-Latino Whites. In addition, they may be more likely to face the added stress of racial discrimination because of the greater percentage of members with African ancestry. In fact, a previous study reported that darker skinned Latinos experience more racial discrimination than lighter skinned Latinos [40].

The above finding provides evidence of within-groups differences among the U.S. Latino population, and is consistent with the results of a previous study that identified differences between the groups in cardiovascular risks and on several indicators of subclinical cardiovascular disease [4]. These results underline the fact that the Latino groups living in the United States are a heterogeneous population, thus should not be treated as a single, or uniform group. These results have implications both in the design and execution of public policies for minorities living in the U.S., and can help our understanding of the contributors to health disparities.

It was expected that social support would moderate the relationship between chronic stress, depressive symptoms and MetS in part because Latinos have been characterized as having high levels of social support from family [41], and several studies have reported positive mental and physical health outcomes as a consequence of family social support [42,43]. However, this prediction was not supported by the analyses, possible again due to restriction in the range on social support. Most of the Latino sample reported high levels of social support, suggesting that this variable probably may moderate these associations when comparing Latinos to other groups. Furthermore, social support may be one area where Latinos are accurately characterized together as a single group.

In this study, we also aimed to determine if inflammation might mediate the association between psychological variables and Mets. However, in light of the lack of association between psychological variables and any of the inflammatory markers, no analysis of mediation was conducted. Our results did indicate that two of the three inflammatory markers, IL-6 and CRP, were related to risk of MetS as independent contributors in the overall Latino sample. Although some slight differences in the main effect of inflammatory markers in each of the Latino groups, this is consistent with previous studies that demonstrated the role of systemic inflammation as a risk factor for MetS [44]. CRP has been identified as a major player in the pathological process associated with MetS as CRP is associated with insulin resistance, endothelial dysfunction and impaired fibrinolysis [45]. Systemic inflammation can lead to clinical and biochemical manifestation of MetS [46]. Therefore it is important for future studies on the role of inflammation among Latinos to test for possible sub-group differences before accepting either positive or negative results.

The MESA dataset provided an excellent opportunity to test these hypotheses because of the large U.S. Latino groups sampled, and the strength of the constructs measured. However, we faced some problems with some variables of interest. With respect to the depression items, despite high internal consistency, it is possible that the language of the interview (English or Spanish) might have produced slight differences in the comprehension or the meaning of the items. This variable was not coded in the dataset and as a consequence, our analyses could not be adjusted by this variable. Also, MESA does not allow disentangling the Central/South Americans group, which is a heterogeneous cluster that includes people from different national origins and different sociodemographic profiles. This heterogeneity makes inferences and conclusions drawn from these results difficult to generalize to other persons from these countries. Another limitation is that inflammatory markers were measured only once and since several factors can influence their concentrations, causal relationship between variables of interest and inflammation cannot be inferred.

Finally, the temporal association of psychological variables and MetS cannot be inferred due to the cross-sectional design. The associations between psychological variables, MetS components, and inflammatory response are essentially dynamic and bidirectional, and although as a strategy we chose to study how psychological variables may be related with MetS as an outcome, we acknowledge that for future studies, it will be necessary to take advantage of the longitudinal study design in order to test whether the psychosocial variables at baseline predict the incidence of MetS at subsequent times.
